# Feasibility of a pulsed multiphase contrast media injection protocol in head and neck computed tomography angiography: a systematic retrospective study

**DOI:** 10.7717/peerj.20216

**Published:** 2025-11-25

**Authors:** Xinghao Yang, Zheng Li, Junyao Cai, Lei Wu, Jingyue Zhang, Yihong Wu, Hua Yao, Shengkun Yuan, Yantong Tao, Kunrong Yu, Shufeng Zhu, Yuan Fang, Dongxu Wang

**Affiliations:** 1 Departments of CT, The Second Affiliated Hospital of Qiqihar Medical University, Qiqihar, Heilongjiang, China; 2 Medical Technology College, Qiqihar Medical University, Qiqihar, Heilongjiang, China

**Keywords:** CTA, Head and neck, Contrast-media, Pulsed multi-phase injection

## Abstract

**Background:**

This study aimed to evaluate the efficacy and value of a pulsed multiphase contrast media injection protocol in head and neck computed tomographic angiography (CTA).

**Information and Methodology:**

A total of 522 patients who underwent head and neck CTA at the Second Hospital of Qiqihar Medical University between March 1, 2022, and March 31, 2024, were reviewed. After excluding 174 patients, 348 were included in the analysis. All scans were performed using a GE Revolution computed tomography (CT) scanner (256-row detector). In the conventional group (*n* = 179), patients received 60 mL of contrast agent followed by 40 mL of saline. In the pulse group (*n* = 169), patients received an initial 35 mL of contrast agent, followed by alternating injections of five mL contrast agent and five mL saline across four additional stages, and concluded with 40 mL of saline. Images were post-processed using a GE workstation with curved planar reformation (CPR), maximum intensity projection (MIP), and volume rendering (VR). CT values, contrast-to-noise ratio (CNR), and signal-to-noise ratio (SNR) were measured for target arteries and veins. Subjective image quality was assessed using a five-point scale.

**Results:**

There were no significant differences in CT values, CNR, or SNR between the conventional and pulse groups. However, the pulse group showed significantly lower CT values in the subclavian vein (SCV) and superior vena cava (SVC) compared to the conventional group (*P* < 0.001). Both groups achieved image quality scores of three or higher, but a statistically significant difference in subjective image quality was observed (*P* < 0.001). Inter-rater agreement was moderate in the conventional group (Kappa = 0.573) and substantial in the pulse group (Kappa = 0.684). Radiation dose analysis revealed a significant reduction in the pulse group (*P* < 0.001), with mean dose-length product (DLP) and effective dose (ED) reduced by 67.58 mGy cm and 0.16 mSv, respectively.

**Conclusion:**

The pulsed multiphase contrast agent injection protocol improves image quality by reducing venous contrast residue while simultaneously lowering radiation exposure and contrast agent usage.

## Introduction

In recent years, with advancements in medical imaging techniques, computed tomographic angiography (CTA) has become the first-line diagnostic modality for evaluating acute ischemic stroke and cerebrovascular aneurysms (You et al., 2015). As a minimally invasive imaging modality with relatively few contraindications (Roberts et al., 2013), head and neck CTA is the preferred diagnostic tool for acute stroke patients due to its faster scan times (Volders et al., 2019).

However, with the widespread application of head and neck CTA (Cabral Frade et al., 2022), some problems of head and neck CTA have also emerged in clinical work. On the one hand, in clinical practice, the conventional two-phase bolus contrast agent injection protocol used for head and neck CTA often results in excessive contrast agent concentration in non-target vessels, compromising target vessel visualization. The artifacts generated by the left/right brachiocephalic vein are the most serious. These venous artifacts increase the difficulty for physicians to perform image post-processing. The CT value of vessels in the CTA image is influenced by both the iodine delivery rate (IDR) and the X-ray energy (tube voltage) (Bae, 2010). The iodine delivery rate (mg I/s) is determined by both the contrast concentration (mg I/mL) and the iodinated contrast injection flow rate (mL/s). Therefore, diluting the concentration of the contrast agent is an effective method to decrease venous CT values and to reduce the occurrence of venous artifacts. It has been demonstrated that the use of a multiphase dual-flow contrast-saline mixed injection protocol in coronary computed tomography angiography (CCTA) significantly reduces the beam-hardening artifact caused by the superior vena cava and the right ventricle (Cao et al., 2009). However, this contrast-saline mixed injection protocol has not yet been systematically investigated in head and neck CTA. The pulsed-multiphase contrast media injection protocol enables the mixed injection of contrast media and saline by alternately delivering the two substances. This study evaluates its clinical utility in head and neck CTA.

On the other hand, the ionizing radiation generated during CTA examinations is garnering growing concern, as it is considered one of the risk factors for cancer development (Demircioğlu et al., 2024). Reducing the ionizing radiation produced during the imaging procedures is one of the current challenges that need to be solved.

In this study, we first systematically evaluated the effects of a pulsed-multiphase contrast injection protocol in head and neck CTA under low kilovolt (kV) imaging parameters. We observed changes in image quality, contrast agent dose, and radiation dose, providing evidence for selecting contrast injection protocols in head and neck CTA.

## Method and Materials

### Research object

We retrieved data on 522 patients who underwent head and neck CTA at our hospital from March 1, 2022, to March 31, 2024, and excluded 174 patients who did not meet the established criteria. All enrolled patients underwent only head and neck CTA examinations. All data were uniformly retrieved and recorded between April 10, 2024, and May 31, 2024. Inclusion criteria: (1) Age >18 and ≤80 years; (2) patients without a history of carotid artery and its branch surgeries, as well as without a history of head and neck cancers or benign tumors. Exclusion criteria: (1) Patients with severe cardiac, hepatic, or renal insufficiency; (2) pregnant or lactating women; (3) patients with a history of iodine allergy; (4) patients with metal implants, where severe metal artifacts are present in the images. Following examination and approval, the institution designated the standardized operation plan on March 25, 2023 and decided to implement the pulsed-multiphase contrast agent injection protocol for all subsequent head and neck CTA cases, with patient grouping being time-limited. The patients at our hospital from March 1, 2022, to March 25, 2023, were assigned to the conventional group, all having utilized the two-phase contrast agent injection protocol. Patients at our hospital from March 26, 2023, to March 31, 2024, who all received the pulsed-multiphase contrast agent injection protocol, were assigned to the Pulse Group. Before March 25, 2023, patients undergoing head and neck CTA examinations at our hospital were administered a conventional two-phase contrast agent injection protocol. After the policy was established on March 25, 2023, all patients were switched to a pulsed multi-phase contrast agent injection protocol. Patients were grouped based on the time periods they visited our hospital for examinations, as different injection protocols were applied during different time periods.

**Figure 1:**
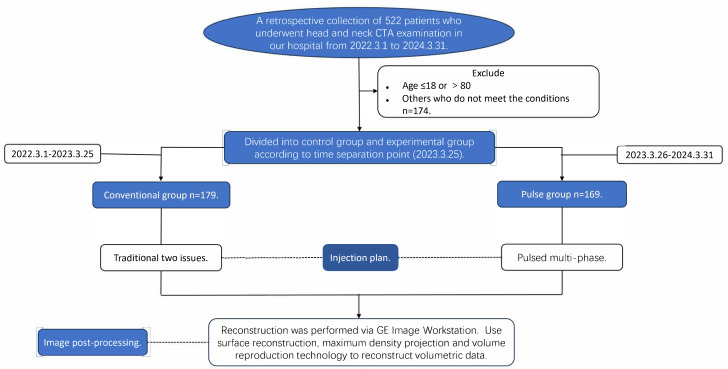
Study flowchart for participating patients.

The study received ethical approval (Lenko Review (2023-01) No. 1) from the Ethics Committee of the Second Affiliated Hospital of Qiqihar Medical University (No. 37, West Zhonghua Road, Jianhua District, Qiqihar, China) on March 9, 2023. This study adhered to the Declaration of Helsinki. Since this was a retrospective analysis, the Ethics Committee permitted the waiver of informed consent requirement; nonetheless, it was emphasized that the confidentiality of patients’ personal information should be maintained. The specific study flow chart is shown in Fig. 1.

### Image acquisition

All patients were scanned using the Revolution™ computed tomography (CT) scanner (GE Healthcare Life Sciences, Waukesha, WI, USA). The scanner is calibrated every morning before it is used. Recalibration is performed in the event of special circumstances, such as system exit. And all imaging data was retained for at least five years. Head and neck CTA imaging protocols: The patient was placed in the supine position. The temporal subtraction method was employed. The initial scan range is as follows: Scanning extends from the aortic arch (AA) to the cranial vertex. Initiating the scan requires a 5-second delay prior to commencement. The parameters for the CTA scanning are as follows: The tube voltage was set to 120 kV for the conventional group and 100 kV for the pulse group in CTA scanning. The tube currents for both groups were configured for automatic adjustment, with values ranging from 150 to 400 mA. The collimator width was 64 × 0.6 mm, with a pitch of 10.992:1 and a tube rotation speed of 0.5 s/rot. All other scanning parameters and contrast media were consistent across both groups. Both patient groups underwent head and neck CTA scans after applying the test-bolus technique. The drug administered was Iopromide (Ultravist 370, 370 mg I/mL; Bayer Schering Pharma) at a volume of 15 mL and an injection speed of four mL/s. A 15 mL saline flush was subsequently administered. A 15-mL saline flush was also used. Scanning of the same layer commenced after a 10-second delay. Monitoring at the bifurcation of the common carotid artery, which is situated at the level of the C4/C5 intervertebral disc, was performed using a region of interest (ROI), with scans every 2 s. Scanning was halted after the peak contrast concentration of the layer was reached, and a time-density curve was then plotted. ROI refers to the area of the target vessel delineated by a circle for treatment or CT value measurement purposes. The peak value of the time-density curve corresponds to the CT value of the peak iodine concentration within the ROI. Additionally, the time-axis value at the peak corresponds to the time point (*t*_*p*_) when peak iodine concentration occurs. The arterial-phase scanning delay time (*T*) is calculated using the formula: *T* = *t*_*p*_ + 2 − plain scanning time. Contrast injection was performed concurrently with the scan. A plain scan without iodine-containing contrast from the AA to the cranial vertex was performed first (scan duration: 1.5−2.5 s), followed by an enhancement scan (scan duration: 1.5−2.5 s), with a delay interval of *T* seconds between the two scans. All scans were performed without breath-holding.

**Figure 2:**
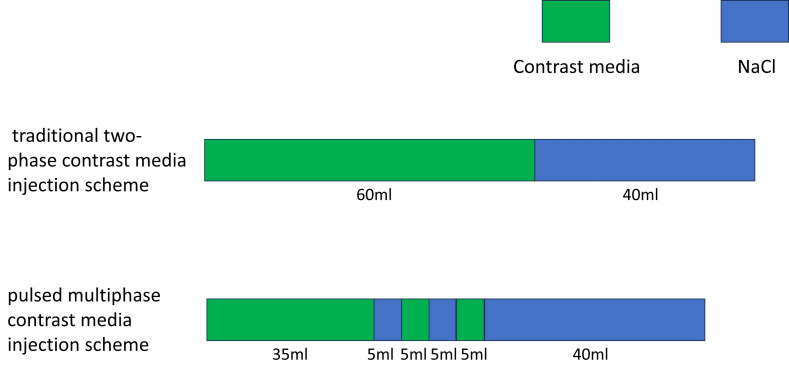
Schematic diagram of the injection protocol.

The contrast agent was injected using a high-pressure double-barreled injector (Urich INJECT CT Motion™; Ulrich Medical, Ulm, Germany). Before the injection, a 24-gauge indwelling needle was inserted into the superficial vein or the median cubital vein of the patient’s right forearm. Injecting 20 mL of saline in advance assessed that the catheter was unobstructed. The infusion rate was set at five mL/s. In the contrast injection program, the conventional group was administered the two-phase injection protocol. The first phase involved the injection of 60 mL of contrast, followed by a second phase that injected 40 mL of saline. The flow rates for the contrast agent and the normal saline were both five ml/s. The total injection time was 20 s. Conversely, the pulse group was administered a pulsed multi-phase contrast injection protocol. In this group, the first phase injected 35 mL of contrast, followed by the second phase with five mL of saline. From the third to the fifth phase, the contrast agent and saline were alternately injected to achieve mixed injection of them. The injection volume of the contrast agent or saline was five mL per phase. Inject 40 mL of saline solution at the final stage. The flow rate was five mL/s, and the total injection duration was 19 s. The contrast agent or saline was injected continuously at each stage. In the first phase, a pure contrast agent was injected to ensure that the target blood vessels are clearly visible. The total injection time in the first stage was 7 s. From phase two to phase five, a mixture of contrast agent and saline was injected to maintain vascular contrast and clear residual contrast agent from the brachiocephalic veins. The injection time at this stage is 4 s. Finally, 35 ml of saline was injected to eliminate any remaining contrast agent more thoroughly. The injection time for the final stage was 8 s. Before conducting this study, we validated the feasibility of the low-kilovoltage, low-contrast-dose scanning protocol using animal models. New Zealand rabbits were randomly divided into three groups: Group A was scanned at 120 kV with a contrast agent dose of 2.5 ml/kg, Group B at 100 kV with a contrast agent dose of 2.5 ml/kg, and Group C at 100 kV with an 80% reduced contrast agent dose (2.5 ml/kg × 80%). The results of the experiment demonstrated that the low contrast agent injection protocol effectively reduced the CT values of LSVC under low-kilovoltage scanning conditions, while producing images of comparable quality to those obtained in Group A, which used higher kV and contrast agent doses (Li et al., 2022). Before modifying the injection protocol, we used a two-phase bolus injection technique. This often resulted in excessive beam-hardening artefacts in the subclavian vein or superior vena cava of patients, which adversely affected image quality. Based on preliminary experimental studies conducted on animals, we redesigned a pulsed, multi-phase contrast agent injection protocol aimed at reducing the occurrence of beam-hardening artefacts. Comparisons of the injection regimens and the parameters of the patients in conventional group and pulse group are detailed in Table 1 and Fig. 2.

**Table 1:** Contrast injection protocols.

Group	Tube voltage	Tube current (automatic adjustment)	Total contrast agent volume (mL)	Injection protocol	Total volume of normal saline (mL)	Flow rate
Conventional group	120 Kv	150–400 mA	75 mL	Conventional two-phase	40 mL	5 mL/s
Pulse group	100 Kv	150–400 mA	60 mL	Pulsed multiphase	55 mL	5 mL/s

### Image post-processing and data analysis

CTA data was reconstructed using the GE Healthcare’s Advantage Workstation. The three-dimensional volume data was reconstructed using techniques such as curved planar reformation (CPR), maximum intensity projection (MIP), and volume rendering (VR). The reconstruction parameters were as follows: the slice thickness for all images was set to 0.625 mm, with a slice interval of 0.625 mm. The window width (WW) was set to 600 HU, and the window level (WL) was set to 100 HU.

Using the double-blind method, two experienced radiologists independently measured the CT values of the AA, the bilateral common carotid arteries (CCA) at the level of the bifurcation of the CCA, the bilateral vertebral arteries (VA) also at the level of the bifurcation of the CCA, the C2 segment of the internal carotid artery (C2), the M1 segment of the middle cerebral artery (M1), the subclavian vein (SCV), and the superior vena cava (SVC). The aforementioned vessels’ CT values were measured independently on the Picture Archiving and Communication System workstation. Use the circular measurement tool in the workstation to measure the CT values of the aforementioned blood vessels, one by one. When measuring CT values, the vascular walls and small calcifications should be excluded. The CT values of the target vessels were recorded, and the average value was used as the mean CT value of the vessel. If one side of an artery was occluded and could not be measured, the value from the contralateral side was used as a substitute. The mean vessel CT values were determined based on the CT values of the AA, CCA, VA, M1 and C2. The SNR was equal to the ratio of the mean CT value of blood vessels between background noise. The CNR was equal to the ratio of mean CT value of blood vessels—background CT value between background noise. The standard deviation of the CT values measured in the trachea at the level of the first cervical vertebra was regarded as background noise, whereas the CT value of the soft tissue positioned behind the trachea at that same level was considered the background CT value. Subjective scoring was conducted by two radiologists (physician A with 15 years of experience and physician B with 35 years of experience in head and neck CTA). Two physicians scored the imaging data in a double-blind manner, and the image sequences of all patients were randomly presented to the two physicians. Physicians were instructed to score the images based on their personal preferences. Referring to the scoring criteria in the study by Tawfik et al. (2012), we have re-established a similar scoring system consisting of three components. These are residual contrast in the brachiocephalic vein ((5) No residual contrast in the vein and the surrounding tissues are clearly displayed; (4) minimal residual contrast in the vein that does not increase vascular contrast; (3) moderate residual contrast in the vein that is only visible within the vessel; (2) residual contrast in the vein that affects the surrounding tissues, but does not impair diagnostic imaging; and (1) residual contrast in the vein that severely affects the surrounding tissues and bone tissues are not properly displayed) and vascular sharpness ((5) Perfect vascular contour boundaries; (4) clear structural contour boundaries; (3) moderate structural contour boundaries; (2) blurred contours; (1) significantly blurred structural contours; and subjective image noise or graininess ((5) no perceptible image noise; (4) Below-average image noise; (3) average image noise and slight graininess; (2) Above-average image noise and moderate graininess; (1) significantly unacceptable noise level). The final score is presented according to Schimeller L’s five-point system.The scoring criteria were based on the 5-point scale utilized in the study by Schimmöller et al. (2013), as presented in Table 2.

**Table 2:** Scoring criteria Schimmöller L five-point method.

Score	Evaluation criterion
5	High contrast, high resolution, no noise, no artifact (1) No residual contrast in the vein, and the surrounding tissues are clearly displayed (2) Perfect vascular contour boundaries (3) No perceptible image noise
4	Good contrast, less artifacts, does not affect the diagnosis (1) Minimal residual contrast in the vein that does not increase vascular contrast (2) Clear structural contour boundaries (3) Below-average image noise
3	There are artifacts and can still meet the diagnosis (1) Moderate residual contrast in the vein that is only visible within the vessel (2) Moderate structural contour boundaries (3) Average image noise and slight graininess
2	Poor contrast, artifacts affect diagnosis (1) Residual contrast in the vein that affects the surrounding tissues, but does not impair diagnostic imaging (2) Blurred contours (3) Above-average image noise and moderate graininess
1	Unable to diagnose (1) Residual contrast in the vein that severely affects the surrounding tissues and bone tissues are not properly displayed (2) Significantly blurred structural contours (3) Significantly unacceptable noise level

The evaluation of the CT radiation dose level was based on two metrics: the dose length product (*DLP*, measured in mGy cm) and the effective dose (*ED*, measured in mSv). The *DLP* is the overall radiation dose associated with a medical examination and was calculated using the formula *DLP* = s*can length*×* volume CT dose index (CTDIvol).* After the examination, the *DLP* can be directly obtained from the CT control panel. The *ED* is determined by the equation *ED* = *DLP*×* k*, with *k* representing the anatomic region-specific conversion coefficient. In the case of CTA scanning for the head and neck, *k* is set at 0.0023 mSv mGy^−1^ cm^−1^ (Schmid, Uder & Lell, 2017).

### Statistical analysis

We analyzed the data using SPSS 26.0 (IBM Corp., Armonk, NY, USA). Normality tests and chi-square tests were conducted on general patient data, measured CT values, SNR, CNR, and radiation doses. For data exhibiting a normal distribution, the results were expressed as the mean ± standard deviation (SD). In contrast, data that did not conform to a normal distribution were reported differently; specifically, these results were presented in terms of the median (interquartile range (IQR)). This method of presentation is often represented as (M (P25, P75)). Categorical data were expressed as frequencies and proportions. When comparing the measurement data of two groups of samples, the independent two-sample *t*-test was employed if the data met the criteria for normal distribution and homogeneity of variance. Conversely, when the data did not conform to a normal distribution, it became necessary to employ the Mann–Whitney U test as a non-parametric alternative. Categorical data was tested with the Pearson chi-square (*χ*^2^) test. We set the significance level at *α* = 0.05. A statistically significant difference was declared when the calculated *P*-value fell below this threshold. An analysis of the disparity in subjective image quality scores between the conventional group and the pulse group was conducted using the Mann–Whitney U test. To assess the agreement of the two physicians’ evaluations of image quality, we used Kappa coefficient. A Kappa value below 0.4 denotes poor agreement, values ranging from 0.4 to 0.75 reflect good agreement, while a value exceeding 0.75 signifies very good agreement.

## Result

### The general information of patients

**Figure 3:**
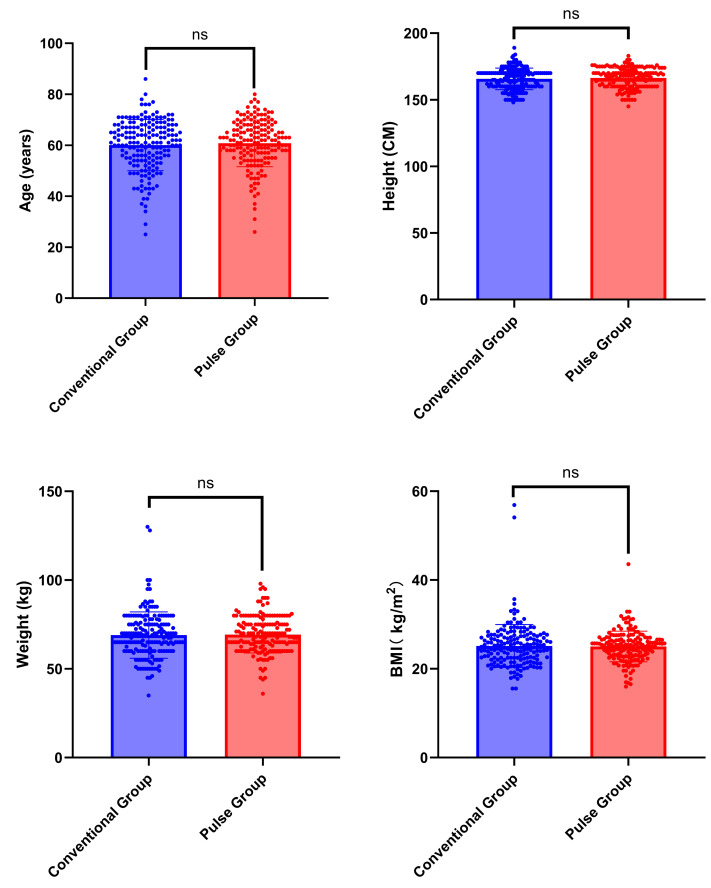
Comparison of general characteristics between conventional group and pulse group. Note: “ns” indicates no statistical significance; “*” indicates *p* < 0.05; “**” indicates *p* < 0.01;“***” indicates *p* < 0.001.

A total of 348 participants were included in this study, with 179 participants assigned to the conventional group and 169 to the pulse group. No statistically significant differences were found in concerning gender, age, height, weight, and BMI when we compared the two groups. The chi-square test was employed in the analysis of gender differences (*χ*^2^ = 0.063, *P* = 0.802). The Mann–Whitney U test was employed for analyzing differences in age, height, weight, and BMI (Z = −0.481, Z= −0.808, Z= −0.682, Z = −0.428; *P* = 0.631, *P* = 0.419, *P* = 0.495, *P* = 0.669). General patient information is summarized in Table 3 and Fig. 3.

### CT Value

**Figure 4:**
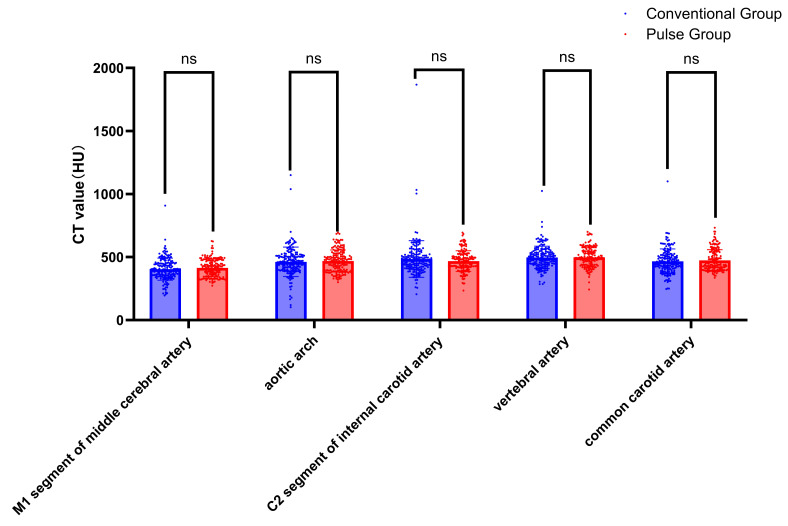
Comparison of CT values of arterial target vessels between conventional group and pulse group. Note: “ns” indicates no statistical significance; “*” indicates *p* < 0.05; “**” indicates *p* < 0.01; “***” indicates *p* < 0.001.

**Figure 5:**
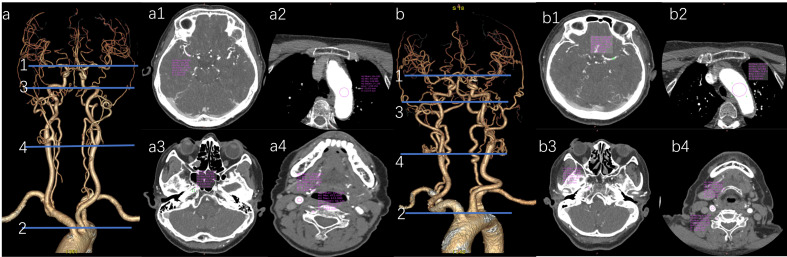
Representative axial CT value measurements of target arteries in both patient groups are presented. (a) Vascular VR image of the head and neck in the pulse group. (a1) The M1’s CT value in the pulse group is 454.05 HU. (a2) The AA’s CT value in the pulse group is 534.21 HU. (a3) The CT value of the C2 in the test group is 552.42 HU. (a4) The CT value of 578.04 HU for the CCA and 569.75 HU for the VA in the test group. (b) Vascular VR images of the head and neck in the conventional group are presented. (b1) The M1’s CT value in the conventional group is 384.06 HU. (b2) The AA’s CT value in the conventional group is 492.15 HU. (b3) The C2’s CT value in the conventional group is 469.03 HU. (b4) The CCA’s CT value in the conventional group is 485.13 HU, while the VA’s CT value is 472.79 HU.

**Figure 6:**
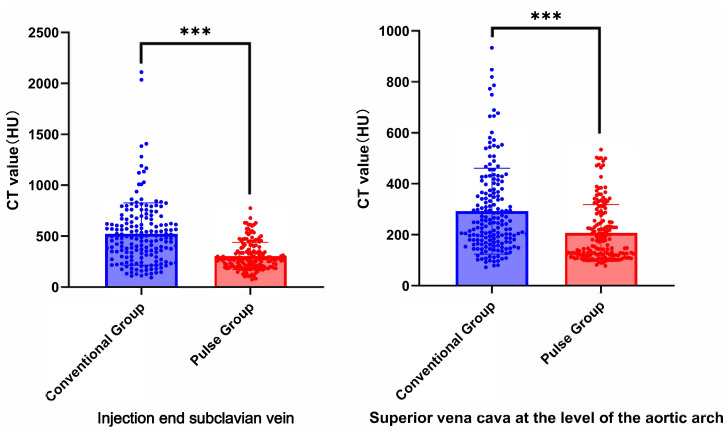
Comparison of the CT values of arterial target vessels between conventional group and pulse group. “ns” indicates no statistical significance. “*” indicates *p* < 0.05; “**” indicates *p* < 0.01; “***” indicates *p* < 0.001.

**Figure 7:**
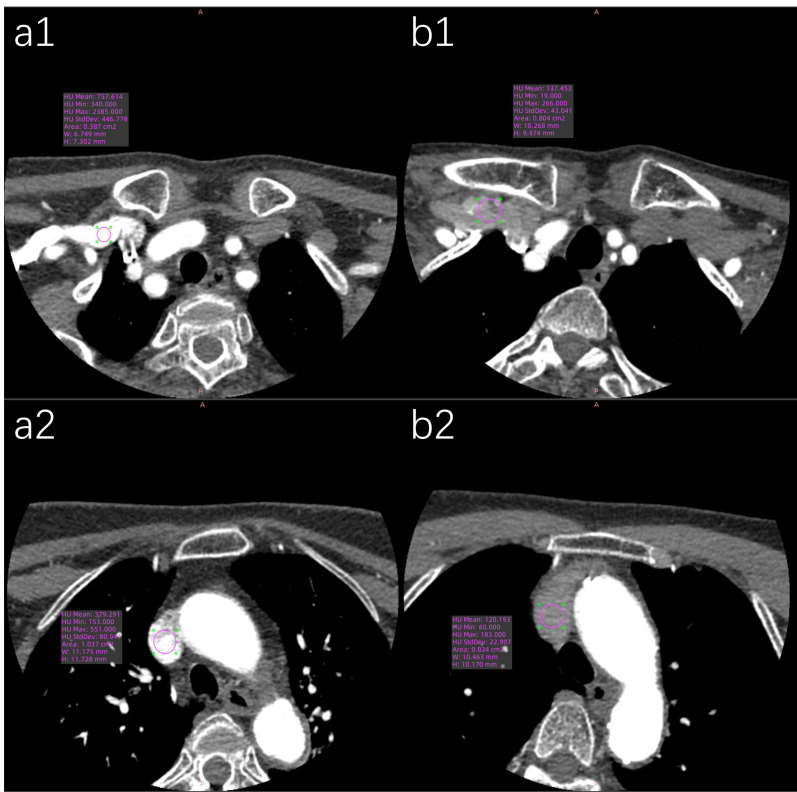
Representative axial CT value measurements of target veins in both patient groups are presented. (a1) The SCV’s CT value in the conventional group is 757.61 HU. (a2) The SVC’s CT value in the conventional group is 379.29 HU. (b1) The SCV’s CT value in the pulse group is 137.45 HU. (b2) The SVC’s CT value in the pulse group is 120.19 HU.

Collect all CT values of target vessels measured by the circular measuring tool in the above workstation. The results showed that the CT values were all above 300 HU. The comparison results of the target vessel CT values between conventional group and pulse group are shown in Table 4, Figs. 4, and 5. The CT attenuation values between the AA, M1, C2, VA, and CCA for the two groups showed no significant differences between groups (*P* > 0.05). The differences in CT values between the SCV and the SVC across both groups were statistically significant (Z = −7.951, *P* < 0.001; Z = −5.539, *P* < 0.001). The pulse group exhibited lower CT values for the SCV and SVC compared to the conventional group, as shown in Figs. 6 and 7.

**Table 3:** The general information of patients.

Parameter	Conventional group	Pulse group	*χ*^2^/*Z*	*P*
Number of cases	179	169	/	/
Gender (Male/Female)	103/76	95/74	*χ*^2^ = 0.063	0.802
Age (years)	61.00 (54.00, 67.00)	62.00 (56.00, 67.00)	*Z* = − 0.481	0.631
Height (cm)	167.00 (160.00, 170.00)	168 (160.00, 172.00)	*Z* = − 0.808	0.419
Weight (kg)	69.00 (60.00, 76.00)	68.00 (62.50, 75.00)	*Z* = − 0.682	0.495
BMI (kg/m^2^)	24.79 (22.29, 27.34)	25.24 (22.91, 26.62)	*Z* = − 0.428	0.669

### SNR and CNR

**Figure 8:**
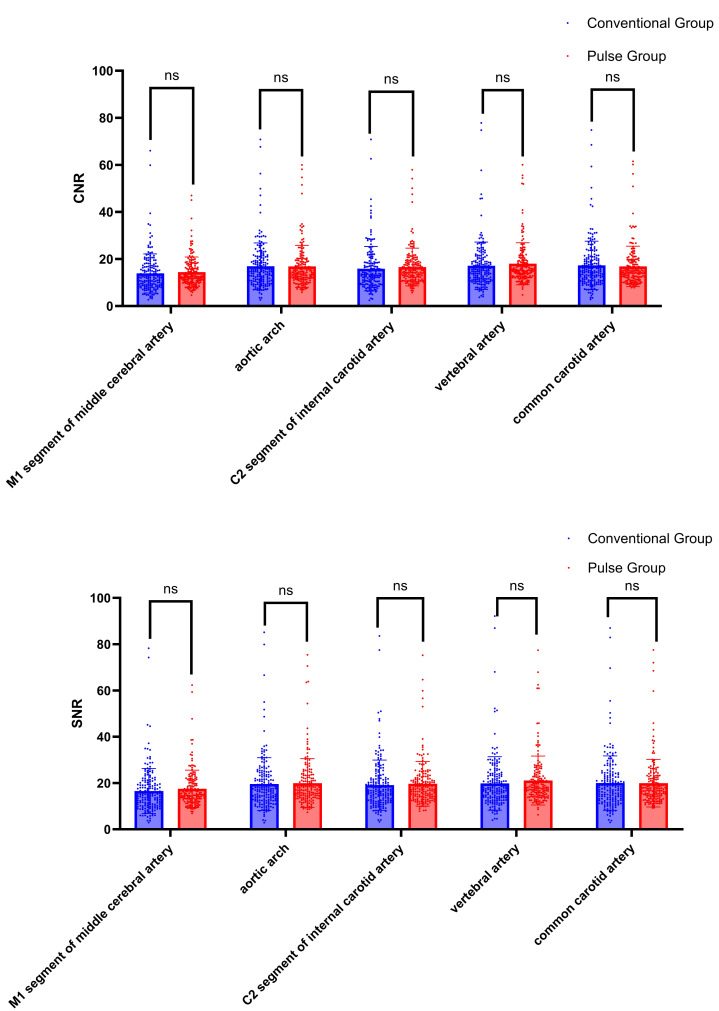
Comparison of CNR and SNR between conventional group and pulse group. Note: “ns” indicates no statistical significance; “*” indicates *p* < 0.05; “**” indicates *p* < 0.01; “***” indicates *p* < 0.001.

The CNR and SNR of the AA, M1, C2, VA, and CCA were compared between the conventional group and the pulse group. The differences were not statistically significant (*P* > 0.05), as shown in Table 5 and Fig. 8.

### Scoring criteria of subjective evaluation of imaging reconstruction

**Figure 9:**
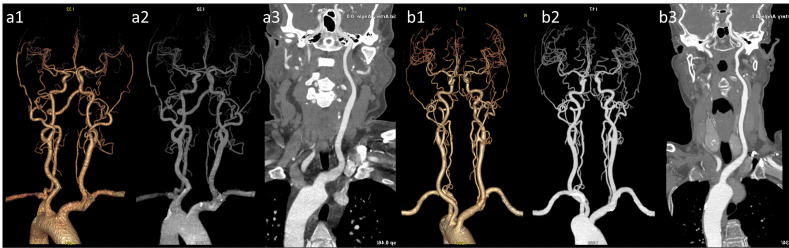
Representative images of VR, MIP, and CPR for both groups are presented. (a1) VR image of the head and neck in the conventional group; (a2) MIP image of the head and neck in the conventional group.) (a3) CPR image of the left common carotid arteries (LCCA) in the conventional group. (b1) VR image of the head and neck in the pulse group; (b2) MIP image of the head and neck in the pulse group; (b3) Curved planar reconstruction image of the LCCA in the pulse group. The images in group (b) exhibit superior quality compared with group (a), featuring a clearer depiction of distal vessels and enhanced contrast.

Two physicians evaluated the image quality of both patient groups, assigning scores ≥ 3 points. Physician A’s subjective ratings for the conventional group and the pulse group were 3.34 ± 0.54 and 3.76 ± 0.78, respectively. Physician B’s subjective ratings for the conventional and pulse groups were 3.23 ± 0.55 and 3.91 ± 0.79, respectively. Physician A’s subjective evaluation results indicate that in the conventional group, 124 cases (69.2%) were rated as three points, 49 cases (27.4%) as four points, and six cases (3.4%) as five points. In the pulse group, 76 cases (45.0%) received a rating of three points, 57 cases (33.7%) as four points, and 36 cases (21.3%) as five points. Physician B’s subjective evaluation results indicate that in the conventional group, 149 cases (83.3%) were rated as three points, 19 cases (10.6%) as four points, and 11 cases (6.1%) as five points. In the pulse group, 61 cases (36.1%) received a rating of three points, 62 cases (36.7%) received four points, and 46 cases (27.2%) received five points. The image quality of the pulse group was higher than that of the conventional group, with statistically significant differences (Physician A: *P* < 0.001; Physician B: *P* < 0.001). The two physicians demonstrated a high level of consistency in their ratings of image quality for the Conventional group and the Pulse group, with a *kappa* value of 0.573 for the Conventional group and 0.684 for the Pulse group, as shown in Table 6 and Fig. 9.

### Comparison of radiation dose

**Figure 10:**
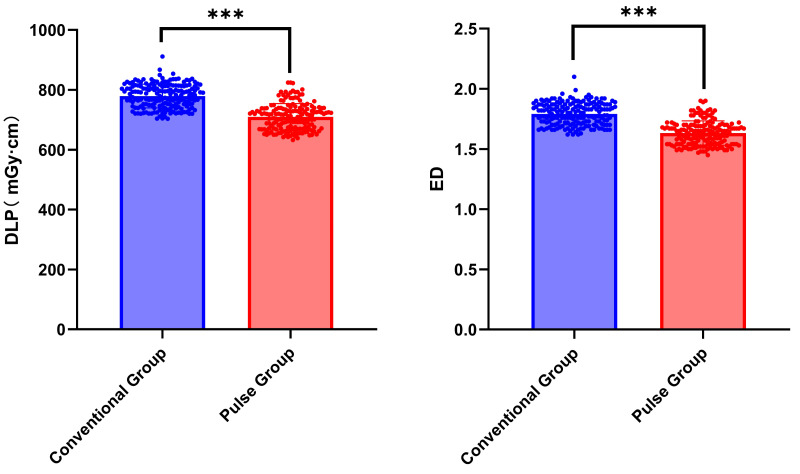
Comparison of Radiation Dose between conventional group and pulse group. Note: “ns” indicates no statistical significance; “*” indicates *p* < 0.05; “**” indicates *p* < 0.01; “***” indicates *p* < 0.001.

The differences in DLP and ED between the conventional group and the pulse group were statistically significant (Z = −11.966, *P* < 0.001; Z = −11.966, *P* < 0.001). Compared with the conventional group, the DLP and ED in the pulse group decreased by approximately 9%. Refer to Table 7 and Fig. 10 for further details.

**Table 4:** Comparison of CT values of target vessels [( 25, 75)].

	Conventional group	Pulse group	*Z*	*P*
M1	397.30 (350.80, 452.70)	413.00 (353.05, 472.95)	−1.156	0.248
AA	458.40 (400.60, 511.00)	473.80 (390.45, 529.00)	−0.554	0.580
C2	469.40 (416.70, 520.20)	472.35 (418.50, 517.83)	−0.661	0.634
VA	489.00 (446.30, 538.40)	492.50 (438.60, 547.70)	−0.477	0.634
CCA	453.60 (414.70, 513.10)	438.50 (401.90, 541.30)	−0.151	0.880
SCV	487.80 (315.90, 664.00)	280.00 (208.90, 353.45)	−7.951	<0.001
SVC	245.40 (171.70, 367.50)	171.60 (120.35, 278.85)	−5.539	<0.001

**Table 5:** Comparison of objective image quality evaluation indicators between conventional group and pulse group.

	Target vessel	Conventional group	Pulse group	Z	*P*
	MCA M1	12.20 (8.68, 16.91)	12.61 (10.30, 17.18)	−1.759	0.079
	AOA	14.67 (10.73, 20.38)	14.38 (11.90, 19.38)	−1.916	0.055
CNR	Petrous Seg.	13.68 (9.59, 18.44)	15.01 (11.37, 18.87)	−0.321	0.748
	VA	15.10 (11.25, 20.01)	15.35 (12.67, 19.58)	−0.649	0.517
	CCA	15.00 (10.80, 20.39)	14.65 (12.08, 19.29)	−1.903	0.057
	MCA M1	14.70 (10.63, 19.76)	15.24 (12.83, 20.73)	−1.365	0.172
	AOA	17.19 (12.60, 23.56)	17.03 (13.87, 22.47)	−1.446	0.148
SNR	Petrous Seg.	16.74 (12.33, 21.82)	17.38 (14.07, 22.05)	−1.655	0.098
	VA	17.32 (13.36, 23.34)	18.03 (15.01, 22.90)	−0.018	0.986
	CCA	17.50 (12.88, 23.60)	17.48 (14.29, 22.29)	−0.416	0.677

## Discussion

In this study, we retrospectively collected the image data and general information of 348 patients. The Conventional group has 179 patients, and the Pulse group has 169 patients. We compared the differences in image quality, contrast agent dose, and radiation dose between the conventional two-phase contrast agent injection protocol and the pulsed multi-phase contrast agent injection protocol under varying tube voltage conditions.

The CT attenuation values of the arteries in all patients exceeded 300 HU. Previous research (Zhou et al., 2023) has demonstrated that a CT attenuation value of 300–400 HU for arterials is sufficient to meet diagnostic requirements. During head and neck CTA examinations, a significant volume of contrast agent frequently is inadvertently delivered to non-target vessels, leading to image quality degradation. This phenomenon is particularly prevalent in the SCV and SVC. Therefore, how reducing the beam hardening artifact caused by the residual intravenous contrast agent is currently one of the key challenges in enhancing the image quality of head and neck CTA. Some scholars have suggested diluting the concentration of contrast media to decrease the incidence of venous contamination (Saade et al., 2017). In the analysis of the target venous CT values in this study, it demonstrated that the CT values of the SCV and SVC in the pulse group decreased by 41.5% and 29.3%, respectively, compared to the conventional group. In head and neck CT angiography (CTA) examinations, the main factors affecting image quality include artifacts caused by high-density structures (such as bones or metal implants) and venous artifacts resulting from the retention of high-concentration contrast agents in veins. Current strategies to enhance image quality primarily focus on two aspects: the application of advanced reconstruction algorithms and the optimization of contrast agent injection protocols. The research by Zhang et al. (2013) demonstrates that the use of low-iodine-concentration contrast agents combined with iterative reconstruction (IR) technology can effectively reduce venous artifacts, improve image quality, and simultaneously reduce radiation dose by 50% and iodine usage by 15.6%. This study benefits from the application of iterative reconstruction technology, which offers greater advantages in radiation dose control, with a radiation dose reduction 41% higher than that of our study. However, the pulsed contrast agent injection protocol employed in our study demonstrated superior performance in reducing iodine dosage, achieving an additional 4.4% reduction compared to Zhang Weilan’s study, while maintaining comparable image quality. Notably, given that not all equipment in clinical practice is equipped with dual-energy scanning capabilities, for devices that only support single-energy scanning, image quality can also be effectively enhanced by adjusting the injection process and adopting the pulsed contrast agent injection protocol. In Cao et al. (2009)’s research, the injection of a contrast-saline mixture using dual-flow technology effectively reduced the beam hardening artifacts caused by high-attenuation contrast in veins and the right ventricle in CCTA examinations. In Cao et al. (2009), Group A followed the conventional two-phase injection protocol, whereas Group B used the dual-flow injection technique. This involved first injecting 50 ml of pure contrast agent, followed by 50 ml of a 6:4 mixture of contrast agent and saline. Although Cao et al. (2009) paper includes a relatively small number of patients, its research methodology is similar to that of our article and the pulsed technique design concept originates from dual-stream technology. Therefore, citing it further validates the feasibility of the pulsed contrast agent injection protocol. The CT attenuation value of veins were decreased by approximately 46%, which aligns with our research findings. In our protocol, the amount of contrast agent was reduced by five mL compared to the protocol of Cao et al. (2009). Our research integrated the Test-Bolus technique with the pulsed multi-phase contrast injection protocol. This approach enables dynamic adjustment of scan-delay time based on patients’ hemodynamic profiles, thereby reducing the residual intravenous contrast agent and minimizing venous artifacts, which further enhances image quality. In our research, we found no statistically significant difference in the target arteries CT attenuation values between the pulse group and the conventional group. Additionally, there were no statistically significant differences in the CNR and SNR between groups. These findings are consistent with the protocol results from the animal model of Overhoff et al. (2020), which showed no significant difference in the arterial enhancement peak between the dual-flow injection protocol at 80 kV and the conventional contrast protocol at 120 kV. In terms of subjective image scoring, the inter-rater agreement of the two physicians is good (kappa in the conventional group = 0.573 and kappa in the pulse group = 0.684). Physician A’s evaluation results: in the conventional group, 69.2% (124/179) were rated three points, while 30.8% (55/179) were rated four or five points; in the pulse group, 45.0% (76/169) were rated three points, and 55.0% (93/169) were rated four or five points. Physician B’s evaluation results: in the conventional group, 83.3% (149/179) were rated three points, and 16.7% (30/179) were rated four or five points; in the pulse group, 36.1% (61/169) were rated three points, whereas 63.9% (108/169) were rated four or five points. Overall, the image quality score for the pulse group was significantly higher to that of the conventional group. The pulsed multi-phase contrast agent injection protocol is based on the dual-flow injection technology. By alternating injections of the contrast agent and saline, the concentration of the contrast agent is effectively reduced. The pulsed injection protocol can effectively reduce the viscosity of iodine contrast agent, accelerate the rate of iodine delivery, decrease the viscosity of the contrast agent in veins, and decrease the beam hardening artifact in the SCV and SVC regions. The reduction of beam hardening artifacts simplifies image post-processing and decreases the time required for radiologists to conduct this process. Currently, there is limited research examining the application of pulsed multiphase contrast media injection protocol in CTA for head and neck imaging. This study is the first to investigate the pulsed multiphase contrast media injection protocol to head and neck CTA examinations. This approach reduces beam hardening artifacts, improves image quality, and decreases the contrast dose. In this study, the pulse group used 60 ml of contrast agent, whereas the conventional group used 75 ml. The amount of contrast agent administered to the pulse group was reduced by 20% compared with the conventional group, resulting in a reduction in incidence of contrast-induced nephropathy (CIN) (Nijssen & Wildberger, 2021). This reduction is particularly significant for patients with certain high-risk factors, including diabetes, dehydration, congestive heart failure, and transient hypertension (Gleeson & Bulugahapitiya, 2004).

**Table 6:** Subjective ratings of image quality for both groups (number of cases).

		Physician A		Physician B	
		Conventional group	Pulse group	Conventional group	Pulse group
One point	Extremely Poor	0	0	0	0
Two points	Poor	0	0	0	0
Three points	Good	124	76	149	61
Four points	Fairly Good	49	57	19	62
Five points	Excellent	6	36	11	46
Score ($\overline{x}~\pm ~s$)		3.34 ± 0.54	3.76 ± 0.78	3.23 ± 0.55	3.91 ± 0.79
*Z*		−5.295		−8.819	
*P*		<0.001		<0.001	

**Table 7:** Comparison of radiation dose.

Item	Conventional group	Pulse group	*Z*	*P*
DLP	782.5 (745.73, 812.57)	714.92 (670.33, 738.72)	−11.966	<0.001
ED	1.80 (1.72, 1.87)	1.64 (1.54, 1.70)	−11.966	<0.001

The implementation of individualized dosing regimens and the extensive use of low-dose contrast agents have significantly decreased the contrast volume used in CTA examinations. The standard package volume for contrast agent commercially available is 100 mL. We are actively engaging with the manufacturer of the contrast agent to recommend the production of a smaller 60-mL packaging size. This change aims to minimize contrast waste associated with traditional high-volume vials and alleviate the economic burden on patients.

The scanning range of head and neck CTA encompasses radiation-sensitive structures that are particularly sensitive to high-dose radiation, such as the thyroid gland and the lens of the eye. Consequently, the radiation exposure experienced by patients during this examination may increase their potential risk of developing cancer. When the human body is exposed to ionizing radiation, X-rays can interact with water molecules to generate hydroxyl radicals. These radicals may then interact with nearby DNA, resulting in strand breaks or base damage. Additionally, X-rays can directly damage DNA, causing cellular damage (Brenner & Hall, 2007). As a result, minimizing the radiation exposure that patients experience is now recognized as a key area of investigation in current studies around the world. Currently, methods for reducing radiation dose include decreasing tube voltage and tube current, employing advanced reconstruction algorithms, and adjusting the detector collimation width, among others (Brady et al., 2021; Flicek et al., 2010). The pulsed multi-phase contrast agent injection protocol, compared with the conventional two-phase injection protocol, reduces the radiation dose by lowering the tube voltage. The study revealed that the patient radiation exposure in the pulse group was approximately 9% lower when compared with that in the conventional group. In Lenfant et al. (2022), under scanning conditions of 100 kV and 120 kV, the former results in a lower radiation exposure. Both DLP and ED are reduced, and the research findings closely align with those presented in this paper. Hower, different results were reported in the study by Namimoto et al. (2012). By decreasing tube voltage, there was a 28.8% reduction in the radiation dose, surpassing the decrease observed reduction in the radiation dose. This discrepancy may be attributed to variations in scanning parameter settings. While we reduced the radiation dose by lowering the tube voltage, their study employed automatic exposure control in conjunction with low-tube-voltage protocols. It is generally believed that reducing tube voltage leads to increased image noise (Del Gaizo et al., 2013). Nonetheless, several research findings have suggested that in low-kilovoltage scanning situations, Compton scattering decreases, while the photoelectric effect becomes more pronounced, and the X-ray photon energy nears the K-edge value for iodine (*K* = 33.2 keV). This results in an increased attenuation value of iodinated contrast agents, ultimately leading to improved vascular contrast enhancement. In the studies by Buls et al., (2015) and Qi et al. (2025), it shown that reducing the tube voltage while decreasing the contrast agent dose does not significantly affect both image quality and diagnostic accuracy. Therefore, the low-contrast-agent dosage and low-kilovoltage scanning technology have broad clinical potential.

This study has certain limitations. Firstly, if a large-sample-size multicenter study can be conducted, then the research results will be more representative and generalizable. Secondly, prospective studies can collect relatively complete data, making it easier to control the influence of confounding factors. Therefore, it is more suitable for this research. Thirdly, in our imaging parameters, we chose automatic exposure control to automatically adjust the tube current. The maximum threshold of tube current modulation (400 mA) is relatively high. We speculate that if this upper limit could be reduced, the radiation dose might consequently decrease. Fourth, the subjective assessment of image quality was conducted by only two physicians, which may have introduced bias. Therefore, we performed a Kappa test on the ratings from the two physicians, and the results indicated good consistency.

## Conclusion

In conclusion, the pulsed multiphase contrast injection protocol demonstrates feasibility in low-kilovoltage head and neck CTA, achieving significant reductions in both contrast agent volume and radiation dose. Furthermore, this approach effectively minimizes venous contrast residue, enhancing overall image quality.

##  Supplemental Information

10.7717/peerj.20216/supp-1 Supplemental Information 1 Raw data

10.7717/peerj.20216/supp-2 Supplemental Information 2 Codebook

## Additional Information and Declarations

### Data Availability

The following information was supplied regarding data availability: The raw data are available in the Supplemental File.

### Clinical Trial Registration

The following information was supplied regarding Clinical Trial registration: Lenko Review (2023-01) No. 1.
