# Supplementary material for: Feasibility of a pulsed multiphase contrast media injection protocol in head and neck computed tomography angiography: a systematic retrospective study
Source: PeerJ. 2025 Nov 25;13:e20216. doi: 10.7717/peerj.20216 (PMC12662063; doi:10.7717/peerj.20216)
Supplement: Supplemental Information 2 [file peerj-13-20216-s002.docx]

**Codebook**

In patient grouping, 1 represents the conventional group, and 2 represents the pulse group.

The conventional group was treated with the traditional two-phase injection protocol, while the pulse group received the pulsed multiphase contrast media injection scheme.

In the gender grouping, 1 represents male and 2 represents female.
